# Supplementary material for: Characterization of the RNA Transcription Profile of Bombyx mori Bidensovirus
Source: Viruses. 2019 Apr 3;11(4):325. doi: 10.3390/v11040325 (PMC6521256; doi:10.3390/v11040325)
Supplement: Supplementary file 1 [file viruses-11-00325-s001.pdf]

viruses

MDPI

Table S1. Primers used for RT-PCR, and RACE.

Primer	Sequence (5′-3′)	Purpose
NS2R	TTCGACTTCATCTGCTGCTTTCCGT	RT-PCR for NS
NS1R	CTTCTTCACCCCAAGAACCATCACCAT	RT-PCR for NS
VPR	TTCATATAAGCGATATCCCCATCCAGT	RT-PCR for VP
PolBR	GGTGCAGGGGTTCCATCTCGATAATC	RT-PCR for PolB
P133R	TGTACTTCTTGTATTGTCTCTCTTCC	RT-PCR for P133
NS3R	TGAAAATCATACTAGATTCACCCCA	RT-PCR for NS3
NS2endR	CTACAGAATCTTAGAGCTCTTTGCAC	5′RACE of NS
NS1endR	CTTAGGAGATAGTTTACACTTTGGAGT	5′RACE of NS
VPendR	GGACTTCCTCCTGGATTAATTCTAGC	5′RACE of VP
polBendR	ACAATTCACCCTTATAACCAAATGGT	5′RACE of PolB
P133endR	GTACAGATTACTGTTCTAATAGTATTATC	5′RACE of P133
NS3endR	TTCAGAATCATTAATTGATTCTAAAC	5′RACE of NS3
3′NS2F	GGCTGACGATCTCCTCTCTAGAATCA	3′RACE of NS
3′NS1F	TGTATTAGAGGAGCCATTCTTGGATC	3′RACE of NS
3′VPF	GCTCCTGGTGTTGTTATTGGAAGAG	3′RACE of VP
3′polBF	CTCCAGGGTTGTGGTCAGATGATAC	3′RACE of PolB
3′P133F	AGAGATGGAAGATTATCAGGATTTAAG	3′RACE of P133
3′NS3F	GGAAATTGGATAATAGAACAACATATCC	3′RACE of NS3
F2	AAATAAGTTTGTTTGGTTAACATGGC	qRT-PCR
F1	ATTCAACGCTCTCAACCGCCCTTCAAAG	qRT-PCR
R	CGATTCCATTCTTCTTTACAATAACAGG	qRT-PCR
PNS1-2F	GGGGTACCATTTATACTTTAAGCCCATAC AAATAAAGAC	P5/5.5 Promoter for BmBDV
PNS1R	GGAAGCTTTTGAAGGACGGTTGAGAGCG	P5/5.5 Promoter for BmBDV
PNS2R	GGAAGCTTTTGACTTCGATTCCATTCTTCT TT	P5/5.5 Promoter for BmBDV

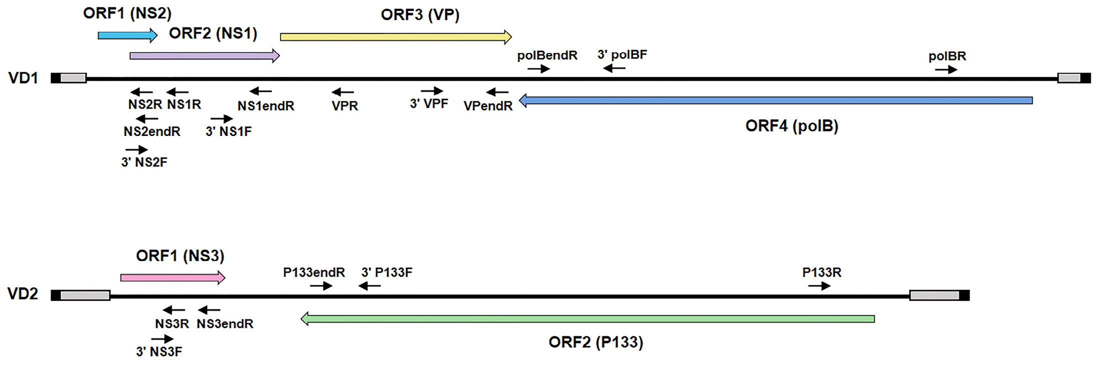

Figure S1. Locations of primers designed for RT-PCR and RACE. Primers are indicated by small arrows.

Table S2. Standard curve equation using qPCR for NS1 and NS2 genes

Gene name	Equations of Standard curves	Amplification Efficiency (E%)	Regression Coefficient (R^2)
NS 1	Y = –3.272*LOG(X) + 38.23	102%	0.995
NS 2	Y = –3.615*LOG(X) + 40.93	90.1%	0.991

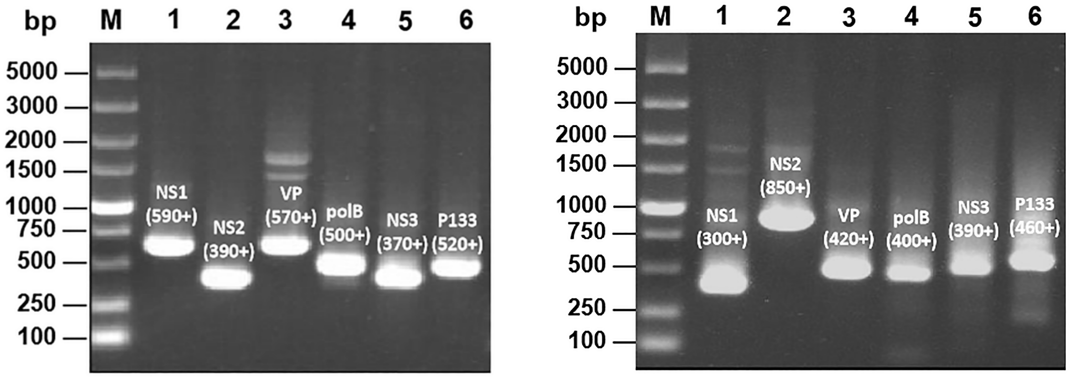

(a) (b)

Figure S2. Analysis of amplicons obtained with different GSPs in 5′ and 3′-RACE (M is 5000 bp marker) of BmBDV transcripts. The observed sizes are in agreement with the expected sizes (values in brackets). (**a**) 5′-RACE of NS1, NS2, VP, polB, NS3, and P133 transcripts; (**b**) 3′-RACE NS1, NS2, VP, polB, NS3, and P133 transcripts.

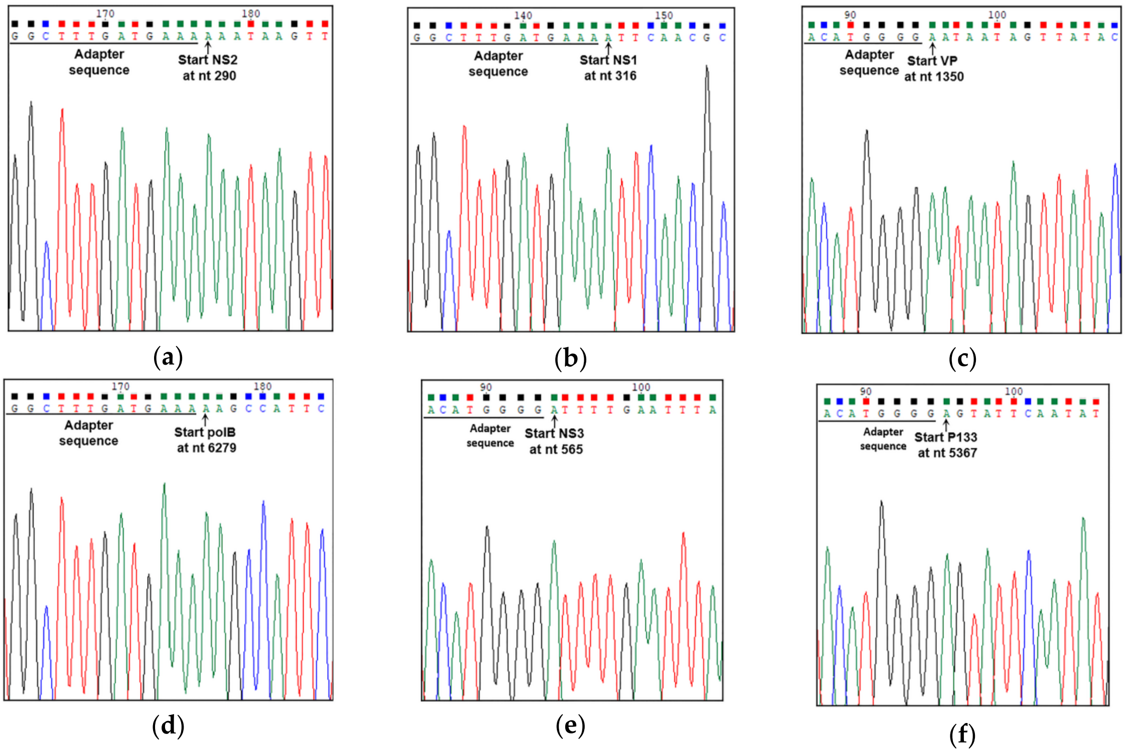

Figure S3. Sequencing results of 5′-RACE. (**a**) Start NS2 transcription. (**b**) Start NS1 transcription. (**c**) Start VP transcription. (**d**) Start polB transcription. (**e**) Start NS3 transcription (**f**) Start P133 transcription.

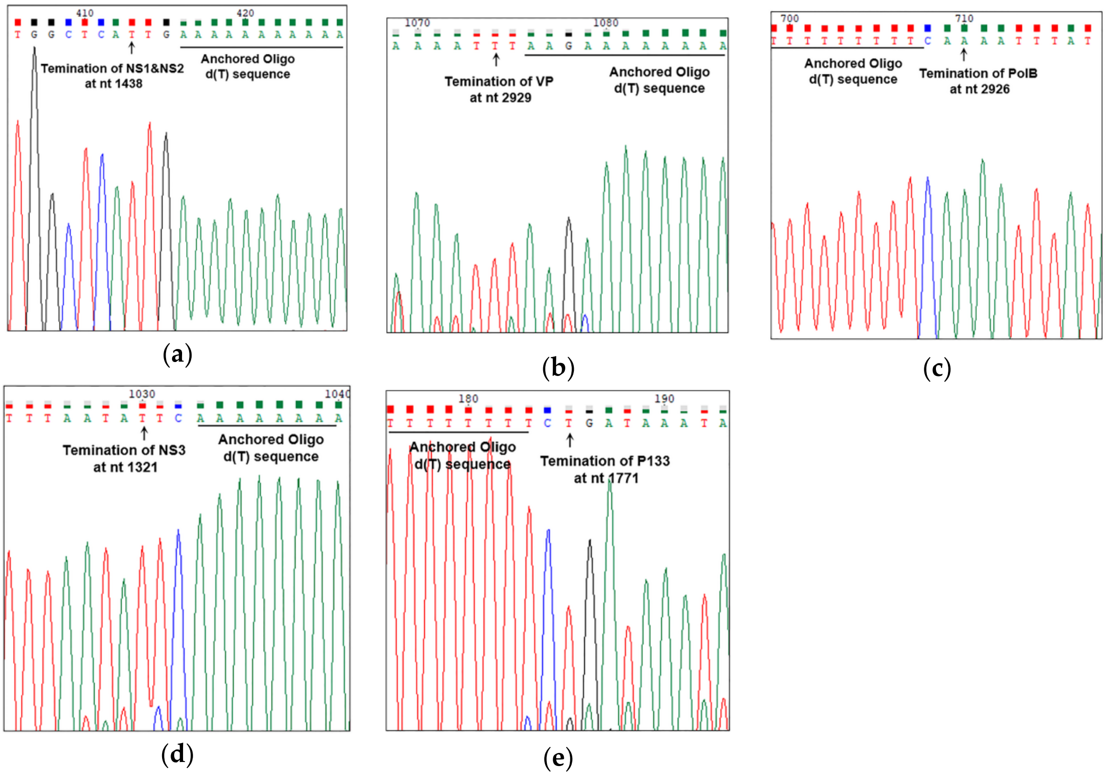

Figure S4. Sequencing results of 3′-RACE. (**a**) Termination of NS2 and NS1 transcripts. (**b**) Termination of VP transcript. (**c**) Termination of PolB transcript. (**d**) Termination of NS3 transcript (**e**) Termination of P133 transcript.

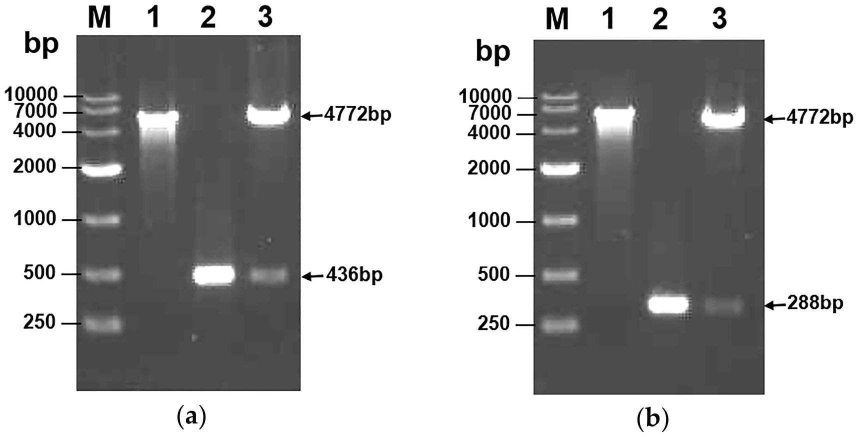

Figure S5. Mapping of recombinant dual-luciferase reporter vectors identified by the restriction enzymes *Kpn*I and *Hind*III. (**a**) Identification of P5 plasmid. M, DNA marker; 1, PGL3-basic; 2, PNS1; 3, P5 plasmid. (**b**) Identification of P5 plasmid. M, DNA marker; 1, PGL3-basic; 2, PNS2; 3, P1 plasmid.

© 2019 by the authors. Submitted for possible open access publication under the terms and conditions of the Creative Commons Attribution (CC BY) license (http://creativecommons.org/licenses/by/4.0/).
